# Di-μ-chlorido-μ-(dimethyl sulfide)-bis­{dichlorido[(dimethyl selenide-κ*Se*)(dimethyl sulfide-κ*S*)(0.65/0.35)]niobium(III)}(*Nb*—*Nb*)

**DOI:** 10.1107/S1600536812033673

**Published:** 2012-08-11

**Authors:** Masatoshi Matsuura, Takashi Fujihara, Akira Nagasawa, Seik Weng Ng

**Affiliations:** a Department of Chemistry, Graduate School of Science and Engineering, Saitama University, Shimo-Okubo 255, Sakura-ku, Saitama 338-8570, Japan; b Comprehensive Analysis Center for Science, Saitama University, Shimo-Okubo 255, Sakura-ku, Saitama 338-8570, Japan; c Department of Chemistry, University of Malaya, 50603 Kuala Lumpur, Malaysia; d Chemistry Department, Faculty of Science, King Abdulaziz University, PO Box 80203 Jeddah, Saudi Arabia

## Abstract

The dinuclear compound, [Nb_2_Cl_6_(C_2_H_6_S)_1.7_(C_2_H_6_Se)_1.3_], features an Nb^III^=Nb^III^ double bond [2.6878 (5) Å]. The mol­ecule lies on a twofold rotation axis that passes through the middle of this bond as well as through the bridging dimethyl sulfide ligand. The Nb^III^ ion exists in an octa­hedral coordination environment defined by two terminal and two bridging Cl atoms, and (CH_3_)_2_Se/(CH_3_)_2_S ligands. The (bridging) ligand lying on the twofold rotation axis is an ordered (CH_3_)_2_S ligand, whereas the terminal ones on a general position are a mixture of (CH_3_)_2_Se and (CH_3_)_2_S ligands in a 0.647 (2):0.353 (2) ratio (the methyl C atoms are also disordered).

## Related literature

For background to this study, see: Cotton *et al.* (1985 ▶); Kakeya *et al.* (2006*a* ▶,*b* ▶). For the synthesis of the principal reactant, see: Tsunoda & Hubert-Pfalzgraf (1982 ▶). For a related structure, see: Babaian-Kibala *et al.* (1991 ▶).
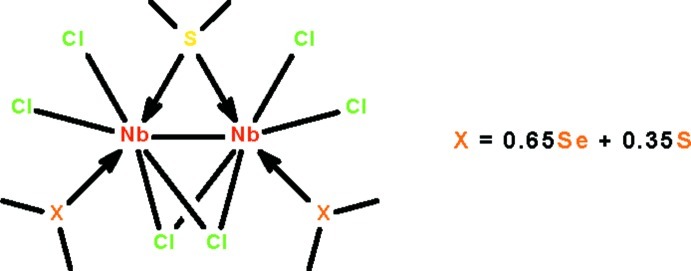


## Experimental

### 

#### Crystal data

[Nb_2_Cl_6_(C_2_H_6_S)_1.7_(C_2_H_6_Se)_1.3_]
*M* *_r_* = 645.87
Orthorhombic, 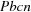

*a* = 13.3314 (11) Å
*b* = 13.5952 (12) Å
*c* = 10.6649 (9) Å
*V* = 1932.9 (3) Å^3^
*Z* = 4
Mo *K*α radiation
μ = 4.63 mm^−1^
*T* = 150 K
0.10 × 0.09 × 0.08 mm

#### Data collection

Bruker APEXII CCD area-detector diffractometer
Absorption correction: multi-scan (*SADABS*; Sheldrick, 1996 ▶) *T*_min_ = 0.655, *T*_max_ = 0.709
10299 measured reflections
2218 independent reflections
1975 reflections with *I* > 2σ(*I*)
*R*_int_ = 0.028

#### Refinement

*R*[*F* ^2^ > 2σ(*F* ^2^)] = 0.023
*wR*(*F* ^2^) = 0.057
*S* = 1.03
2218 reflections
85 parameters
17 restraints
H-atom parameters constrained
Δρ_max_ = 0.61 e Å^−3^
Δρ_min_ = −0.53 e Å^−3^

### 

Data collection: *APEX2* (Bruker, 2008 ▶); cell refinement: *SAINT* (Bruker, 2008 ▶); data reduction: *SAINT*; program(s) used to solve structure: *SHELXS97* (Sheldrick, 2008 ▶); program(s) used to refine structure: *SHELXL97* (Sheldrick, 2008 ▶); molecular graphics: *X-SEED* (Barbour, 2001 ▶); software used to prepare material for publication: *publCIF* (Westrip, 2010 ▶).

## Supplementary Material

Crystal structure: contains datablock(s) global, I. DOI: 10.1107/S1600536812033673/bt5971sup1.cif

Structure factors: contains datablock(s) I. DOI: 10.1107/S1600536812033673/bt5971Isup2.hkl

Additional supplementary materials:  crystallographic information; 3D view; checkCIF report

## Figures and Tables

**Fig. 1.**
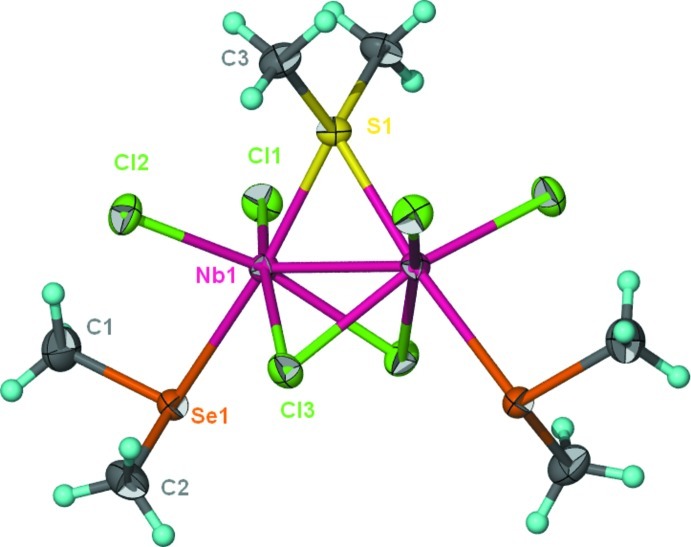
Thermal ellispoid plot (Barbour, 2001) plot of [Nb2Cl6(C2H6Se)1.3(C2H6S)1.7] at the 70% probability level. The disorder is not shown and symmetry-related atoms are not labeled.

## supplementary crystallographic information

### Comment

The chemistry of the lower oxidation states of niobium in discrete complexes remains relatively unexplored. Our research group has already carried out X-ray crystallographic determinations of the complexes of the general formula [Nb_2_Cl_6_*L*_3_] (*L*= tetrahydrothiophene C_4_H_8_S, dimethyl sulfide (Kakeya *et al.*, 2006*a*, 2006*b*). These complexes have a triply bridged face-sharing dioctahedral structure with one thioether as a bridging ligand and two terminal Cl^-^ and thioether. A series of ligand substitution reactions of these complexes with monodentate oxygen donors and substituted phosphanes has been explored. The structures of the face-sharing dioctahedral complexes preserve their original geometry in the case of reaction with monodentate ligands and ligand substitution occurred only at terminal positions (Cotton *et al.*, 1985). We report here the first success in determining the structure of [Nb_2_Cl_6_(C_2_H_6_Se)_1.3_(C_2_H_6_S)_1.7_ (Scheme I), which has selenoether as ligands at terminal positions.

The molecule has dinuclear bridging unit [Nb_2_(µ-Cl)_2_(µ-Me_2_X)] (X = mixture of S, Se) with the terminal Me_2_X ligands in a *trans* orientation to the bridging Me_2_S (Fig. 1). The average Nb—(µ-Cl) and Nb—(µ-Me_2_S) distances fall within the range of those for [Nb_2_(µ-Cl)_2_Cl_4_(µ-Me_2_S)(Me_2_S)_2_], which has the same bridging unit (Kakeya *et al.*, 2006*a*, 2006*b*). The terminal Nb—Cl lengths are shorter than the corresponding distances to the bridging atoms. If the terminal metal-chalcogen bond were purely ionic, the distance should coincide with the sum of ionic radii of metal and chalcogen. Since the ionic radii of the trivalent niobium, Se^2–^ and S^2–^ given in the literature are 0.72 Å, 1.98 Å and 1.84 Å, the bond distances of the metal and chalcogen is 2.70 Å for Se^2–^ and 2.56 Å for S^2–^. We find that the difference between bond lengths and sum of the those radii is smaller in the title compound than in [Nb_2_Cl_6_(Me_2_S)_3_]. This difference is ascribed to the influence of the covalency of the metal-chalcogen interactions. Other geometrical parameters also lie within the same ranges as in analogous dinuclear niobium complexes (Kakeya *et al.*, 2006*a*, 2006*b*).

### Experimental

All the reactions were carried out under a dry argon atmosphere by using standard Schlenk tube techniques. [Nb_2_Cl_6_(Me_2_S)_3_] was prepared by a literature method (Tsunoda & Hubert-Pfalzgraf, 1982). Me_2_Se (0.10 ml, 1.3 mmol) was added to [Nb_2_Cl_6_(Me_2_S)_3_] (200 mg, 0.34 mmol) in CH_2_Cl_2_ (20 ml) and stirred for 1 d at room temperature. The resulting solution was concentrated to dryness to give red purple powders. The crude product was washed with hexane and dried under reduced pressure. A mixture of [Nb_2_Cl_6_(Me_2_S)_3_] and a compound believed to be [Nb_2_Cl_6_(Me_2_Se)_2_(Me_2_S)] was obtained as red purple powders (130 mg, yield 58% identified by ^1^H NMR). The latter compound was recrystallized from CH_2_Cl_2_–hexane (1:4) to give red crystals. ^1^H NMR (CDCl_3_, 300 K): δ 3.33 (6*H*, bridging-Me_2_S), 2.49 (12*H*, terminal-Me_2_Se). ^13^C NMR (300 K, CDCl_3_): δ 30.0 (2 C, bridging-Me_2_S), 14.1 (4 C, terminal-Me_2_S). FAB-MS(nitrobenzyl alcohol matrix): *m*/*z* = 644 [M—Cl]^+^.

As the formulation refined to [Nb_2_Cl_6_(C_2_H_6_Se)_1.3_(C_2_H_6_S)_1.7_], the crystal is probably not reprensentative of the bulk formulation.

### Refinement

The H atoms were placed in calculated positions, with C—H = 0.98 Å for CH_3_, and refined using a riding model, with *U*_iso_(H) = 1.5*U*_eq_ of the carrier atoms.

The chalcogen ligand on the general position is disordered; the atom refined to a 0.647 (2)Se : 0.353S mixture. The Se1–C distances were restrained to 1.95±0.01 Å and the S1'–C distances to 1.80±0.01 Å. The temperature factors of Se1 and S1' were made identical.

The refinement led to an Nb1–Se1 distance of 2.72 Å but a much longer Nb1–S1' distance of 2.79 Å (a normal Nb–S bond is approximately 2.40 Å). Refinement then proceeded by setting the Nb1–S1 (ordered sulfur) and the N1b–S1' (disordered sulfur) bond distances to be within 0.01 Å of each other. The two methyl groups were each split into two components, and the temperature factors of the primed atoms were set to those of the unprimed ones. Additionally, the anisotropic temperature factors were tightly restrained to be nearly isotropic. This model gave distances of 2.420 (1) Å for the ordered atom and 2.543 (6) Å for the disordered atom.

The failure of the Hirshfeld test for the Nb1–Se1 and Nb1–S1' bonds is attributed to the tight restraints imposed on the disordered ligand.

Arising from the refinement, the compound is formulated as [Nb_2_Cl_6_(C_2_H_6_Se)_1.3_(C_2_H_6_S)_1.7_].

### Crystal data

**Table:**

[Nb_2_Cl_6_(C_2_H_6_S)_1.7_(C_2_H_6_Se)_1.3_]	*F*(000) = 1238
*M**_r_* = 645.87	*D*_x_ = 2.219 Mg m^−^^3^
Orthorhombic, *P**b**c**n*	Mo *K*α radiation, λ = 0.71073 Å
Hall symbol: -P 2n 2ab	Cell parameters from 3921 reflections
*a* = 13.3314 (11) Å	θ = 2.9–28.5°
*b* = 13.5952 (12) Å	µ = 4.63 mm^−^^1^
*c* = 10.6649 (9) Å	*T* = 150 K
*V* = 1932.9 (3) Å^3^	Block, red
*Z* = 4	0.10 × 0.09 × 0.08 mm

### Data collection

**Table:**

Bruker APEXII CCD area-detector diffractometer	2218 independent reflections
Radiation source: Bruker TXS fine-focus rotating anode	1975 reflections with *I* > 2σ(*I*)
Bruker Helios multilayer confocal mirror monochromator	*R*_int_ = 0.028
Detector resolution: 8.333 pixels mm^-1^	θ_max_ = 27.5°, θ_min_ = 2.1°
φ and ω scans	*h* = −15→17
Absorption correction: multi-scan (*SADABS*; Sheldrick, 1996)	*k* = −16→17
*T*_min_ = 0.655, *T*_max_ = 0.709	*l* = −12→13
10299 measured reflections	

### Refinement

**Table:**

Refinement on *F*^2^	Primary atom site location: structure-invariant direct methods
Least-squares matrix: full	Secondary atom site location: difference Fourier map
*R*[*F*^2^ > 2σ(*F*^2^)] = 0.023	Hydrogen site location: inferred from neighbouring sites
*wR*(*F*^2^) = 0.057	H-atom parameters constrained
*S* = 1.03	*w* = 1/[σ^2^(*F*_o_^2^) + (0.0281*P*)^2^ + 2.0744*P*] where *P* = (*F*_o_^2^ + 2*F*_c_^2^)/3
2218 reflections	(Δ/σ)_max_ = 0.001
85 parameters	Δρ_max_ = 0.61 e Å^−^^3^
17 restraints	Δρ_min_ = −0.53 e Å^−^^3^

### Fractional atomic coordinates and isotropic or equivalent isotropic displacement parameters (Å^2^)

**Table:**

	*x*	*y*	*z*	*U*_iso_*/*U*_eq_	Occ. (<1)
Nb1	0.468766 (18)	0.233109 (19)	0.36981 (2)	0.01461 (9)	
Se1	0.43903 (16)	0.38286 (10)	0.54126 (16)	0.0169 (2)	0.647 (2)
S1'	0.4374 (8)	0.3682 (6)	0.5299 (8)	0.0169 (2)	0.353
Cl1	0.57325 (6)	0.16964 (6)	0.53247 (6)	0.02378 (16)	
Cl2	0.31130 (5)	0.16593 (6)	0.42645 (7)	0.02496 (17)	
Cl3	0.38916 (5)	0.33927 (5)	0.20578 (6)	0.02154 (16)	
S1	0.5000	0.08504 (7)	0.2500	0.0173 (2)	
C1	0.3683 (5)	0.3155 (6)	0.6751 (7)	0.0327 (8)	0.647 (2)
H1A	0.3411	0.2533	0.6434	0.049*	0.647 (2)
H1B	0.3133	0.3570	0.7053	0.049*	0.647 (2)
H1C	0.4148	0.3021	0.7442	0.049*	0.647 (2)
C2	0.3289 (7)	0.4616 (8)	0.4702 (10)	0.0240 (12)	0.647 (2)
H2A	0.2946	0.4234	0.4051	0.036*	0.647 (2)
H2B	0.3562	0.5219	0.4333	0.036*	0.647 (2)
H2C	0.2812	0.4785	0.5367	0.036*	0.647 (2)
C1'	0.3660 (7)	0.3227 (8)	0.6639 (10)	0.0327 (8)	0.353
H2D	0.2942	0.3330	0.6490	0.049*	0.3532 (16)
H2E	0.3863	0.3584	0.7396	0.049*	0.3532 (16)
H2F	0.3791	0.2524	0.6751	0.049*	0.3532 (16)
C2'	0.3338 (14)	0.4497 (16)	0.489 (2)	0.0240 (12)	0.353
H3D	0.2715	0.4241	0.5255	0.036*	0.3532 (16)
H3E	0.3273	0.4534	0.3981	0.036*	0.3532 (16)
H3F	0.3470	0.5156	0.5231	0.036*	0.3532 (16)
C3	0.4005 (2)	0.0020 (2)	0.2071 (3)	0.0246 (6)	
H3A	0.3415	0.0398	0.1811	0.037*	
H3B	0.3831	−0.0393	0.2793	0.037*	
H3C	0.4227	−0.0398	0.1376	0.037*	

### Atomic displacement parameters (Å^2^)

**Table:**

	*U*^11^	*U*^22^	*U*^33^	*U*^12^	*U*^13^	*U*^23^
Nb1	0.01621 (14)	0.01427 (14)	0.01335 (13)	−0.00034 (9)	−0.00035 (9)	0.00066 (9)
Se1	0.0202 (2)	0.0139 (6)	0.0167 (4)	−0.0004 (4)	0.0026 (3)	−0.0051 (3)
S1'	0.0202 (2)	0.0139 (6)	0.0167 (4)	−0.0004 (4)	0.0026 (3)	−0.0051 (3)
Cl1	0.0265 (4)	0.0263 (4)	0.0185 (3)	0.0031 (3)	−0.0059 (3)	0.0021 (3)
Cl2	0.0209 (4)	0.0286 (4)	0.0254 (4)	−0.0064 (3)	0.0031 (3)	−0.0001 (3)
Cl3	0.0244 (4)	0.0224 (4)	0.0179 (3)	0.0073 (3)	0.0008 (3)	0.0027 (3)
S1	0.0198 (5)	0.0147 (5)	0.0174 (5)	0.000	−0.0024 (4)	0.000
C1	0.045 (2)	0.0319 (19)	0.0213 (18)	−0.0017 (16)	0.0105 (15)	0.0018 (15)
C2	0.0259 (18)	0.022 (3)	0.024 (4)	0.0093 (15)	−0.0041 (18)	0.003 (2)
C1'	0.045 (2)	0.0319 (19)	0.0213 (18)	−0.0017 (16)	0.0105 (15)	0.0018 (15)
C2'	0.0259 (18)	0.022 (3)	0.024 (4)	0.0093 (15)	−0.0041 (18)	0.003 (2)
C3	0.0249 (15)	0.0219 (16)	0.0270 (16)	−0.0055 (12)	−0.0045 (13)	0.0007 (12)

### Geometric parameters (Å, º)

**Table:**

Nb1—Cl2	2.3676 (7)	C1—H1A	0.9800
Nb1—Cl1	2.3863 (7)	C1—H1B	0.9800
Nb1—S1	2.4204 (8)	C1—H1C	0.9800
Nb1—Cl3	2.5039 (7)	C2—H2A	0.9800
Nb1—Cl3^i^	2.5140 (7)	C2—H2B	0.9800
Nb1—S1'	2.543 (6)	C2—H2C	0.9800
Nb1—Nb1^i^	2.6878 (5)	C1'—H2D	0.9800
Nb1—Se1	2.7650 (10)	C1'—H2E	0.9800
Se1—C1	1.941 (6)	C1'—H2F	0.9800
Se1—C2	1.968 (6)	C2'—H3D	0.9800
S1'—C1'	1.825 (9)	C2'—H3E	0.9800
S1'—C2'	1.822 (9)	C2'—H3F	0.9800
Cl3—Nb1^i^	2.5140 (7)	C3—H3A	0.9800
S1—C3	1.801 (3)	C3—H3B	0.9800
S1—C3^i^	1.801 (3)	C3—H3C	0.9800
S1—Nb1^i^	2.4204 (8)		
			
Cl2—Nb1—Cl1	101.10 (3)	C3—S1—Nb1^i^	120.94 (10)
Cl2—Nb1—S1	88.07 (2)	C3^i^—S1—Nb1^i^	121.93 (10)
Cl1—Nb1—S1	89.00 (2)	C3—S1—Nb1	121.93 (10)
Cl2—Nb1—Cl3	91.42 (3)	C3^i^—S1—Nb1	120.94 (10)
Cl1—Nb1—Cl3	164.53 (3)	Nb1^i^—S1—Nb1	67.46 (3)
S1—Nb1—Cl3	100.57 (2)	Se1—C1—H1A	109.5
Cl2—Nb1—Cl3^i^	166.23 (3)	Se1—C1—H1B	109.5
Cl1—Nb1—Cl3^i^	90.05 (3)	H1A—C1—H1B	109.5
S1—Nb1—Cl3^i^	100.29 (2)	Se1—C1—H1C	109.5
Cl3—Nb1—Cl3^i^	76.37 (3)	H1A—C1—H1C	109.5
Cl2—Nb1—S1'	87.8 (2)	H1B—C1—H1C	109.5
Cl1—Nb1—S1'	82.5 (3)	Se1—C2—H2A	109.5
S1—Nb1—S1'	169.6 (2)	Se1—C2—H2B	109.5
Cl3—Nb1—S1'	89.0 (3)	H2A—C2—H2B	109.5
Cl3^i^—Nb1—S1'	85.7 (2)	Se1—C2—H2C	109.5
Cl2—Nb1—Nb1^i^	121.15 (2)	H2A—C2—H2C	109.5
Cl1—Nb1—Nb1^i^	120.69 (2)	H2B—C2—H2C	109.5
S1—Nb1—Nb1^i^	56.272 (14)	S1'—C1'—H2D	109.5
Cl3—Nb1—Nb1^i^	57.795 (18)	S1'—C1'—H2E	109.5
Cl3^i^—Nb1—Nb1^i^	57.431 (17)	H2D—C1'—H2E	109.5
S1'—Nb1—Nb1^i^	133.6 (2)	S1'—C1'—H2F	109.5
Cl2—Nb1—Se1	89.33 (4)	H2D—C1'—H2F	109.5
Cl1—Nb1—Se1	82.48 (5)	H2E—C1'—H2F	109.5
S1—Nb1—Se1	170.45 (5)	S1'—C2'—H3D	109.5
Cl3—Nb1—Se1	88.67 (5)	S1'—C2'—H3E	109.5
Cl3^i^—Nb1—Se1	84.11 (4)	H3D—C2'—H3E	109.5
S1'—Nb1—Se1	1.6 (3)	S1'—C2'—H3F	109.5
Nb1^i^—Nb1—Se1	132.31 (4)	H3D—C2'—H3F	109.5
C1—Se1—C2	100.2 (3)	H3E—C2'—H3F	109.5
C1—Se1—Nb1	102.0 (3)	S1—C3—H3A	109.5
C2—Se1—Nb1	104.6 (4)	S1—C3—H3B	109.5
C1'—S1'—C2'	89.8 (9)	H3A—C3—H3B	109.5
C1'—S1'—Nb1	111.5 (5)	S1—C3—H3C	109.5
C2'—S1'—Nb1	113.9 (10)	H3A—C3—H3C	109.5
Nb1—Cl3—Nb1^i^	64.77 (2)	H3B—C3—H3C	109.5
C3—S1—C3^i^	102.3 (2)		
			
Cl2—Nb1—Se1—C1	35.3 (2)	Cl1—Nb1—Cl3—Nb1^i^	89.16 (10)
Cl1—Nb1—Se1—C1	−66.0 (2)	S1—Nb1—Cl3—Nb1^i^	−38.270 (17)
Cl3—Nb1—Se1—C1	126.7 (2)	Cl3^i^—Nb1—Cl3—Nb1^i^	59.87 (2)
Cl3^i^—Nb1—Se1—C1	−156.8 (2)	S1'—Nb1—Cl3—Nb1^i^	145.7 (2)
Nb1^i^—Nb1—Se1—C1	168.8 (2)	Se1—Nb1—Cl3—Nb1^i^	144.13 (4)
Cl2—Nb1—Se1—C2	−68.8 (4)	Cl2—Nb1—S1—C3	16.75 (12)
Cl1—Nb1—Se1—C2	−170.1 (4)	Cl1—Nb1—S1—C3	117.89 (12)
Cl3—Nb1—Se1—C2	22.7 (4)	Cl3—Nb1—S1—C3	−74.34 (12)
Nb1^i^—Nb1—Se1—C2	64.8 (4)	Cl3^i^—Nb1—S1—C3	−152.23 (12)
Cl2—Nb1—S1'—C1'	33.1 (6)	S1'—Nb1—S1—C3	83.3 (13)
Cl1—Nb1—S1'—C1'	−68.4 (6)	Nb1^i^—Nb1—S1—C3	−113.40 (12)
S1—Nb1—S1'—C1'	−33.5 (19)	Cl2—Nb1—S1—C3^i^	−115.09 (12)
Cl3—Nb1—S1'—C1'	124.6 (6)	Cl1—Nb1—S1—C3^i^	−13.95 (12)
Cl3^i^—Nb1—S1'—C1'	−159.0 (7)	Cl3—Nb1—S1—C3^i^	153.82 (12)
Nb1^i^—Nb1—S1'—C1'	165.8 (4)	Cl3^i^—Nb1—S1—C3^i^	75.93 (12)
Cl2—Nb1—S1'—C2'	−66.7 (10)	S1'—Nb1—S1—C3^i^	−48.6 (14)
Cl1—Nb1—S1'—C2'	−168.2 (10)	Nb1^i^—Nb1—S1—C3^i^	114.76 (12)
S1—Nb1—S1'—C2'	−133.3 (13)	Cl2—Nb1—S1—Nb1^i^	130.15 (2)
Cl3—Nb1—S1'—C2'	24.8 (10)	Cl1—Nb1—S1—Nb1^i^	−128.71 (2)
Cl3^i^—Nb1—S1'—C2'	101.2 (10)	Cl3—Nb1—S1—Nb1^i^	39.061 (18)
Nb1^i^—Nb1—S1'—C2'	66.0 (11)	Cl3^i^—Nb1—S1—Nb1^i^	−38.832 (17)
Cl2—Nb1—Cl3—Nb1^i^	−126.58 (2)	S1'—Nb1—S1—Nb1^i^	−163.3 (13)

Symmetry code: (i) −*x*+1, *y*, −*z*+1/2.
